# Recruitment to the pilot phase of Start2quit – lessons learned

**DOI:** 10.1186/1745-6215-12-S1-A120

**Published:** 2011-12-13

**Authors:** Leanne M Gardner, Hazel Gilbert, Irwin Nazareth

**Affiliations:** 1 Research Department of Primary Care and Population Health, University College London, London, UK

## Background

The recruitment of smokers to trials investigating smoking cessation is often difficult and participation rates are low. Programs using reactive recruitment methods reply upon smokers contacting the program in response to an advertised service or treatment. Generally only smokers motivated to quit will respond and thus participation rates for reactive recruitment are less than 5% of the target population. Conversely proactive recruitment, where a service is offered to potential participants directly, can recruit larger proportions and a more diverse range of population. Start2quit, a randomised controlled trial aimed at increasing the use of NHS Stop Smoking Services, will utilize proactive recruitment. We explored response rates and the effects of sending reminder questionnaires in the pilot phase of this study. Furthermore, we compared recruitment in a London-based PCT versus an outside-London-based PCT.

## Methods

In the pilot phase of Start2quit, GP practices were identified through the local Primary Care Research Network in Camden and Oxfordshire. Current smokers aged 16 and over were identified from their medical records and sent an invitation to participate in the study and a baseline questionnaire. Replies were processed three weeks later. Those not responding were sent a reminder invitation letter and questionnaire. Of those responding, eligible smokers were randomized to one of the intervention groups. Replies were processed a further two times at three and six weeks following mail out of the reminder. The main outcome measure was the recruitment rate, i.e. the proportion of eligible participants.

## Results

For the pilot phase, three GP practices from Camden and four GP practices from Oxfordshire were selected for the recruitment of smokers. There was variability in the response rate between GP practices (2.7% to 9.4%), however rates increased in all practices between the first invitation letter and the reminder invitation. The average response rate in Camden (3.2%; London-based PCT) was lower than in Oxfordshire (6.8%; outside-London PCT). An overall a response rate of 5.5% was obtained over the two PCTs.

## Conclusions

We found in the pilot phase of Start2quit that practice location and sending reminder invitation letters and questionnaires can influence recruitment rates. Response rates in London were found to be lower in general and thus other strategies may be required to increase participation in this area. The proactive method used in Start2quit to recruit smokers, identified from their computerised medical records in GP practices, is effective at inviting participants to smoking cessation trials.

